# A promising approach using Fibonacci sequence-based optimization algorithms and advanced computing

**DOI:** 10.1038/s41598-023-28367-9

**Published:** 2023-02-28

**Authors:** H. Tran-Ngoc, T. Le-Xuan, S. Khatir, G. De Roeck, T. Bui-Tien, Magd Abdel Wahab

**Affiliations:** 1 Department of Bridge and Tunnel Engineering, Faculty of Civil Engineering, University of Transport and Communications, Hanoi, Vietnam; 2 Soete Laboratory, Department of Electrical Energy, Metals, Mechanical Constructions, and Systems, Faculty of Engineering and Architecture, Ghent University, 9000 Gent, Belgium; 3 Faculty of Civil Engineering, Ho Chi Minh City Open University, Ho Chi Minh City, Vietnam; 4 Department of Civil Engineering, KU Leuven, 3001 Leuven, Belgium

**Keywords:** Civil engineering, Computational science

## Abstract

In this paper, the feasibility of Structural Health Monitoring (SHM) employing a novel Fibonacy Sequence (FS)-based Optimization Algorithms (OAs) and up-to-date computing techniques is investigated for a large-scale railway bridge. During recent decades, numerous metaheuristic intelligent OAs have been proposed and immediately gained a lot of momentum. However, the major concern is how to employ OAs to deal with real-world problems, especially the SHM of large-scale structures. In addition to the requirement of high accuracy, a high computational cost is putting up a major barrier to the real application of OAs. Therefore, this article aims at addressing these two aforementioned issues. First, we propose employing the optimal ability of the golden ratio formulated by the well-known FS to remedy the shortcomings and improve the accuracy of OAs, specifically, a recently proposed new algorithm, namely Salp Swarm Algorithm (SSA). On the other hand, to deal with the high computational cost problems of OAs, we propose employing an up-to-date computing technique, termed superscalar processor to conduct a series of iterations in parallel. Moreover, in this work, the vectorization technique is also applied to reduce the size of the data. The obtained results show that the proposed approach is highly potential to apply for SHM of real large-scale structures.

## Introduction

During service life, bridges are easily subjected to various damages due to natural impacts (storms, floods, earthquakes, etc.) or human-induced impacts (overload, collision, etc.)^1–5^. In addition, bridges also have their own vibration patterns that possibly cause amplified vibrations when the natural frequencies of the bridges coincide with those of moving vehicles. This mechanical resonance may put bridges in potential danger. Therefore, in recent decades, SHM systems have been widely deployed and captured special attention from the scientific community. The task of SHM systems is to monitor early damages based on measurement data to evaluate the severity of these damages before making timely repair decisions^6–8^.

SHM is mainly based on two main methods: (1) static behaviour-based method and (2) dynamic behaviour-based method^9,10^. While the former employs static responses such as stress, strain, or displacement to assess the structural health condition, the latter relies on dynamic responses such as natural frequencies, mode shapes, or damping ratio. Therefore, dynamic behaviour-based methods are more sensitive to detecting damages occurring in the structures^11^. The performance of the modal identification measurements is essential to build a reliable model for assessing structural health^12^. Experimental measurements can be conducted under ambient and/or artificial excitation. Artificial excitation can be accomplished using an artificial excitation source such as a hammer or a shaker. However, this approach is only suitable for small structures since it is challenging to generate responses large enough to capture the dynamic characteristics of large-scale structures^13^. On top of that, the lowest natural frequencies of large-scale structures are usually outside the frequency band of maximum artificial excitation. Ambient excitation can be produced by wind, micro-seismic, or by passing vehicles. This ambient excitation source is possibly generated randomly at a low cost and does not interfere with the flow of traffic on the bridge^14^.

Over the last decades, numerous OAs have been proposed and successfully applied for a wide range of fields^15–17^. In the SHM field, OAs assist in reducing the deviations between the Finite Element Model (FEM) and measurements. Afterward, the updated model possibly predicts the structural behaviour accurately. SSA is a new OA proposed in 2017^18^ that has recently appeared and immediately gained a lot of momentum. This algorithm is based on the swarming mechanism of Salps to tackle optimization problems. The main advantages of SSA are the capacity of avoiding getting stuck in local minima and storing large optimal solutions. SSA has been well used for recent optimization fields. For instance, Rizk-Allah et al.^19^ combined SSA with a modified Arctan transformation to deal with binary problems. A combination between SSA and K-Nearest Neighbour (KNN) used to look for the optimal solutions of 20 benchmark datasets was proposed in the work of^20^. Faris et al.^21^ employed SSA based on two new wrapper feature selections to deal with optimization problems of 22 UCI (University of California at Irvine) datasets.

Despite the merits of SSA reported in the literature ^18–21^, this algorithm still exposes fundamental shortcomings such as poor global search capacity, an imbalance between exploitation and exploration capacity, and high computational cost. Moreover, like other group-based algorithms, SSA employs suboptimal threshold coefficients to split populations into different groups to seek optimal solutions, which significantly reduces its effectiveness.

For this reason, in this paper, we propose workable solutions to the drawbacks of SSA. First, to deal with unbalanced problems of exploitation and exploration capacity of SSA, we rearrange the number of elements of SSA in the leading group and the following group. Additionally, the elements utilized for exploiting new optimal solutions are provided with acceleration based on the working principle of Particle Swarm Optimization (PSO) to improve the search speed and search space. Last but not least, the most important target in this work focuses on dealing with the drawback of the use of suboptimal threshold coefficients of SSA. Specifically, suboptimal threshold coefficients of SSA are replaced by the golden ratio. It is commonly acknowledged that the golden ratio has demonstrated its optimal ability and can be seen in all kinds of inanimate natural phenomena as well as in human creations. This is the main inspiration to exploit the enormously optimal potential of FS to boost the efficacy of OAs.

Nevertheless, it is commonly acknowledged that to apply OAs for real-world problems, especially for SHM of large-scale structures, apart from the requirement of accuracy, a high computational cost must be solved. To deal with this problem, we employ up-to-date techniques such as superscalar processors and vectorization techniques for OAs. The superscalar processor technique helps to conduct a series of iterations in parallel, whereas the vectorization technique plays a crucial role in reducing the size of data.

The proposed method, namely FSPSOSSA or HSSAPSO (Hybrid SSAPSO), is employed to deal with inverse problems of a real large-scale truss bridge. To compare with FSPSOSSA, other algorithms, namely PSO, Genetic Algorithm (GA), Cuckoo Search (CS), Grey Wolf Optimizer (GWO), SSA, Biogeography-based Optimization (BBO)^22^, Moth-Flame Optimization (MFO)^23^, other improved SSA (ISSA)^24^ are employed.

From the working principle of the FSPSOSSA, some contributions of this work can be drawn as follows:

- Employ the global search capacity of PSO to remedy the shortcomings and improve the effectiveness of traditional SSA.
- Propose applying the golden ratio to rearrange the populations of SSA. This is a vital premise to improve the efficiency of all group-based algorithms using thresholds like SSA.
- The effectiveness of FSPSOSSA is demonstrated by not only dealing with inverse problems of a real large-scale structure, but also by comparing it with other well-known algorithms.
- Propose a new approach applying advanced techniques such as superscalar processor and vectorization techniques to OAs. For this contribution, the computational time is extremely reduced. This approach is highly potential to apply OAs to tackle real problems.

## Methodology

Although in recent decades, numerous OAs have been proposed and successfully used for theoretical models, employing OAs to deal with real-world problems is still challenging. To achieve that goal, two problems including accuracy and calculation time must be solved. Therefore, in this section, we come up with workable solutions to improve the accuracy and reduce the computational time of the SSA^18^.

### SSA

**Figure 1:**
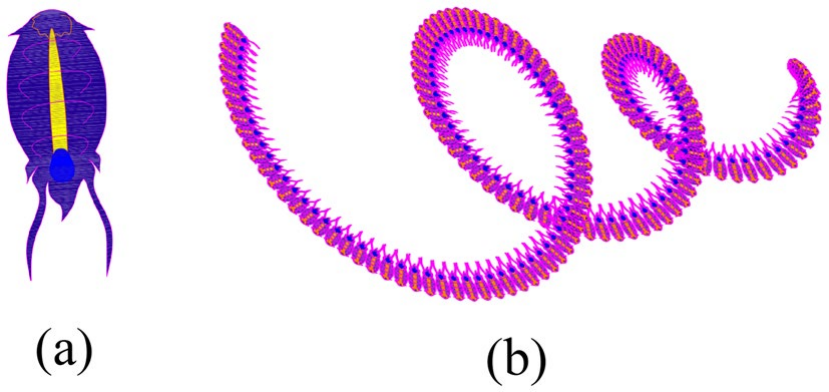
(**a**) Salp and (**b**) Salp Chain.

Salp of the family Salpidae is a barrel-shaped, as a semi-transparent marine animal as shown in Fig. 1a.

During the process of seeking food, Salps often float together in a form of a chain depicted in Fig. 1b. Each Salp group consists of one leading element (at the beginning of each chain) and the following ones. The position of the leader is identified using Eq. (1)^18^.

1 $${x}_{j}^{1}=\left\{\begin{array}{c}{P}_{j-1}+{c}_{1}*\left({U}_{j-1}-{L}_{j-1}\right)*{c}_{2}+{L}_{j-1} {c}_{3}\ge 0 \\ {P}_{j-1}-{c}_{1}*\left({U}_{j-1}-{L}_{j-1}\right)*{c}_{2}+{L}_{j-1} {c}_{3}<0 \end{array}\right.$$

where ${x}_{j}^{1}$ is the position of the leader in the $j$ dimension; ${P}_{j-1}$ is the global optimum (the best solution obtained so far); ${U}_{j-1}$ and ${L}_{j-1}$ are the upper bound and the lower bound of the search space in the $j-1$ dimension, respectively. ${c}_{2}$ and ${c}_{3}$ are random coefficients with their values in a range of [0,1], whereas ${c}_{1}$ is calculated using Eq. (2)^18^.

2 $${c}_{1}=2*{e}^{-{\left({\frac{4*k}{K}}\right)}^{2}}$$

where $k$ is the current iteration and $K$ is the total number of iterations.

To follow the leader, followers utilise Eq. (3)^18^:

3 $${x}_{j}^{i}=\frac{1}{2}({x}_{j}^{i}+{x}_{j}^{i-1})$$

With $i\ge 2$; ${{x}_{j}^{i-1}\mathrm{ and }x}_{j}^{i}$ indicate the position of Salp *i* −1th and *i*th.

Although SSA has proven its ability to solve optimization problems reported in the literature, it still has the following major disadvantages:

- SSA uses only one leader to discover new optimal solutions, whereas the remaining elements only serve as storage. This throws off the balance between exploitation and exploration capacity.
- SSA depends crucially on the movement of the leader. In the last step when $k$ (the current iteration) is close to $K$ (the maximum iteration) as shown in Eq. (2), the jump step of the leader is small. This not only causes a slow convergence and increases the search time, but also reduces the accuracy of the obtained results.
- SSA employs suboptimal threshold coefficients to split populations into different groups to seek optimal solutions, which significantly reduces its effectiveness.

### Fibonacci sequence (FS) and the golden ratio

FS was proposed by Leonardo Fibonacci and has become popular in the 19th century. FS and the golden ratio show up in our world in diverse forms. In nature, the golden ratio can be observed in flowers, snail shells, ammonite shells, and so forth. Likewise, many organs of the human being also show up in the golden ratio, for instance, the number of petals of flowers, the spiral of the ear, the spirals of DNA, the forearm concerning the hand, and so on. In terms of human creation, the golden ratio is applied to architecture such as the Parthenon, the Eiffel tower, the Pyramids of Giza, and so forth.

**Figure 2:**
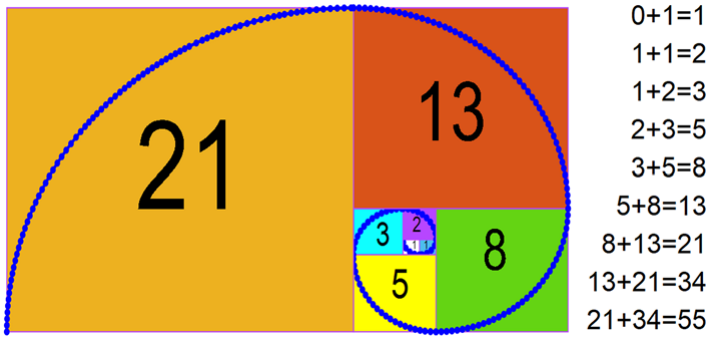
FS.

FS is built from the rule of a sequence of numbers, in which the number after is the summation of two continuous numbers before, which is described in Fig. 2.

Based on the FS, the golden ratio $\varphi$ is built using Eq. (4).

4 $$\varphi =\frac{{b}_{z}}{{b}_{z}+{b}_{z+1}} \mathrm{or }\varphi =\frac{{b}_{z}+{b}_{z+1}}{{b}_{z}}$$

5 $${b}_{z+1}={b}_{z-1}+{b}_{z}$$

${b}_{z-1}; {b}_{z};\mathrm{and }{b}_{z+1}$ are number *z* – 1th;*z*th; *z* + 1th of FS; respectively.

For example:

$$\varphi \approx \frac{8}{5+8}\approx \frac{13}{8+13}\approx \frac{21}{13+21}\approx \frac{34}{21+34}\approx \frac{55}{34+55}\approx \dots \approx 0.618$$

Or

$$\varphi \approx \frac{5+8}{8}\approx \frac{8+13}{13}\approx \frac{13+21}{21}\approx \frac{21+34}{34}\approx \frac{34+55}{55}\approx \dots \approx 1.618$$

### Vectorisation technique

In mathematics, the vectorisation of a matrix is a linear transformation that transforms the matrix into a column vector. In the other words, the vectorisation of matrix ${B}_{n\times m}$, named vec ($B$), is the $nm$×1 column vector is acquired by making a stack of one column on top of others:

6 $${vec \left(B\right)=[{b}_{\mathrm{1,1}},\dots ,{b}_{n,1 }{,b}_{\mathrm{1,2}},\dots ,{b}_{n,2 }, {b}_{1,m },\dots , {b}_{n,m}]}^{T}$$

${b}_{i,j}$ denotes $B$(i,j), whereas the superscript $T$ represents the transpose. Vectorisation indicates, through coordinates, the isomorphism ${R}^{n\times m}$: = ${R}^{n} \otimes {R}^{m} \cong {R}^{nm}$. For instance, for the 3 × 3 matrix $$B=\left[\begin{array}{ccc}{a}_{1}& {b}_{1}& {c}_{1}\\ {a}_{2}& {b}_{2}& {c}_{2}\\ {a}_{3}& {b}_{3}& {c}_{3}\end{array}\right]$$, the vectorisation is:

$$\mathrm{vec }\left(B\right)=\left[\begin{array}{c}{a}_{1}\\ {a}_{2}\\ \begin{array}{c}{a}_{3}\\ {b}_{1}\\ \begin{array}{c}{b}_{2}\\ \begin{array}{c}{b}_{3}\\ {c}_{1}\\ \begin{array}{c}{c}_{2}\\ {c}_{3}\end{array}\end{array}\end{array}\end{array}\end{array}\right]$$

### Superscalar processor

**Figure 3:**
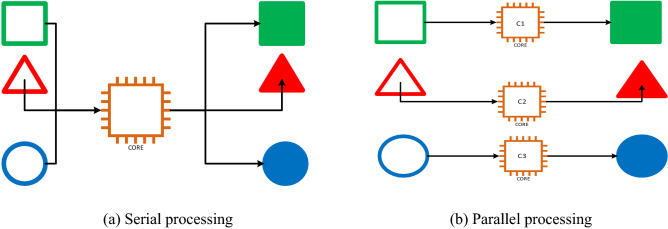
Serial processing and parallel processing.

Parallel processing is a computation method that runs two or more Central Processing Units (CPUs) to process separate parts of an overall task. Dividing different parts of a task between multiple processors plays a vital role in reducing computational time. Any system with more than one CPU can do parallel processing. A multi-core processor is an Integrated Circuit (IC) chip that contains two or more processors for better performance, and reduced power consumption. These multi-core setups aim to install multiple separate processors on the same computer. Most computers can have between two and four cores. However, exploiting this feature to make the calculation process faster and more efficient, i.e. making codes faster and more efficient, for specific problems remains a challenge for researchers. Therefore, in this study, we propose to exploit the potential of parallel processors to reduce the computational time of the OAs. That increases the applicability of OAs to solve real-world problems. Figure 3 depicts the differences between serial processing and parallel processing. The serial processor completes one task at a time by using only one processor (core), whereas parallel processors can accomplish many tasks using two or more processors.

In this paper, the superscalar processor is employed to reduce the computational cost that facilitates the SHM process. Specifically, the superscalar processor (parallel processing) is used to run two or more CPUs simultaneously to process separate parts of an overall task.

### FSPSOSSA

To deal with the shortcomings of SSA, in this section, effective solutions will be proposed, including the following main characteristics:

- To generate a balance between exploitation and exploration capacity, the number of elements of SSA in the leading group and the following group is rearranged. The elements are split into three groups. The first one is the leading group using 30% of the population instead of solely using one leader as SSA. The following one includes 2 groups. The first one using 40% of the population is to store the optimal solutions, and in the last one, 30% of the population is assigned an additional weight $w$ derived from PSO to speed up the velocity of elements.
- With SSA, the movement of elements uses coefficient ${c}_{3}$ with a threshold equal to 0 (Eq. 1). In this study, coefficient ${c}_{3}$ of SSA is replaced by the golden ratio with three thresholds as follows:
  - Type 1: upward movement (${c}_{3}$ > 0.618).
  - Type 2: downward movement (${c}_{3}$ < − 0.618).
  - Type 3: mutant generators [$-0.618\le {c}_{3}\le 0.618$].

Each Salp chain is split into 3 groups: Leading Group (LD), Follower Group 1 (FG1), and Follower Group 2 (FG2).

For LD: $$i=\frac{4*m}{10}$$

7 $${x}_{j}^{i}=\left\{\begin{array}{c}{P}_{j-1}+{c}_{1}*\left({U}_{j-1}-{L}_{j-1}\right)*{c}_{2}+{L}_{j-1} {c}_{3}>0.618 \left(1\right)\\ {x}_{j-1}^{i}+{v}_{j}^{i} -0.618\le {c}_{3}\le 0.618 \left(2\right)\\ {P}_{j-1}+{c}_{1}*\left({U}_{j-1}-{L}_{j-1}\right)*{c}_{2}+{L}_{j-1} {c}_{3}<-0.618 \left(3\right)\end{array}\right.$$

8 $${v}_{j}^{i}={w*v}_{j-1}^{i}+{c}_{1}^{\mathrm{^{\prime}}}*{r}_{1}*\left({P}_{j-1}-{x}_{j-1}^{i}\right)+{c}_{2}^{\mathrm{^{\prime}}}*{r}_{2}*\left({p}_{j-1}^{i}-{x}_{j-1}^{i}\right)$$

$m$ indicates the number of population;${x}_{j}^{i}, {x}_{j}^{i-1}, {v}_{j}^{i}, {v}_{j-1}^{i}$ denote position and velocity of the Salp leader *i*th at the *j*th and *j* − 1th iteration; respectively; ${c}_{1}^{\mathrm{^{\prime}}}$, ${c}_{2}^{\mathrm{^{\prime}}}$, ${r}_{1}$ and ${r}_{2}$ are the cognition learning and social learning factor; and random numbers (0 < ${r}_{1},{r}_{2 }$< 1), respectively, $w$ is the inertia weight parameter, ${p}_{j-1}^{i}$ represents the local best of particle $i$ at *j* − 1th iteration.

For FG1: $$i:\frac{4*m}{10}\div\frac{7*m}{10}$$

9 $${x}_{j}^{i}=\frac{1}{2} ({x}_{j}^{i}+{x}_{j}^{i-1})$$

For FG2: $$i:\frac{7*m}{10}\div m$$

10 $${x}_{j}^{i}={x}_{j-1}^{i}+{v}_{j}^{i}$$

**Figure 4:**
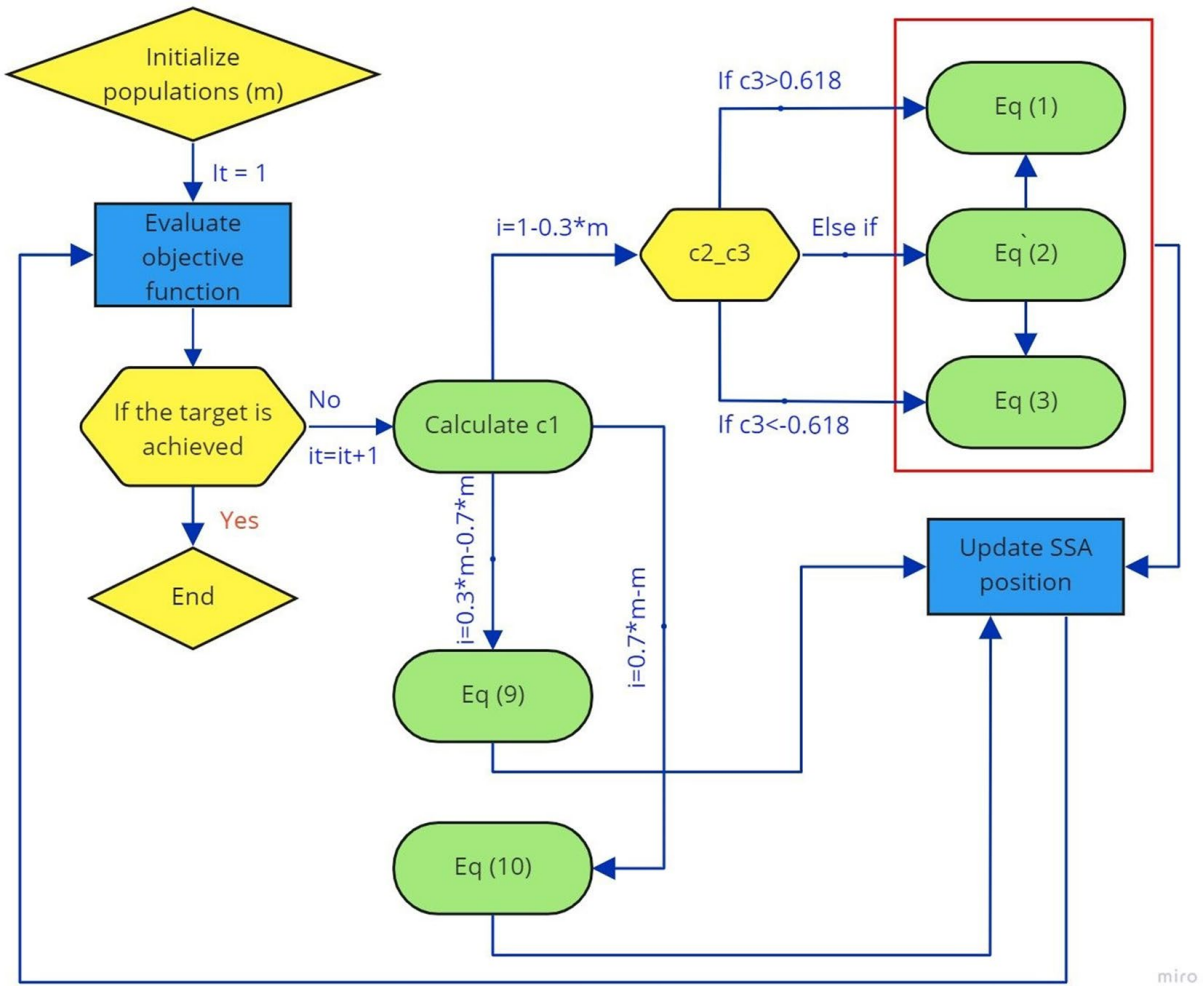
The working principle of FSSSAPSO.

The working principle of FSSAPSO is depicted in Fig. 4.

FSPSOSSA is utilized to deal with inverse problems. Based on objective functions, FSPSOSSA is employed to identify uncertain parameters that can exactly represent the behaviours of the structures.

### Application of the proposed approach to SHM of a real large-scale truss bridge

#### Bridge description

**Figure 5:**
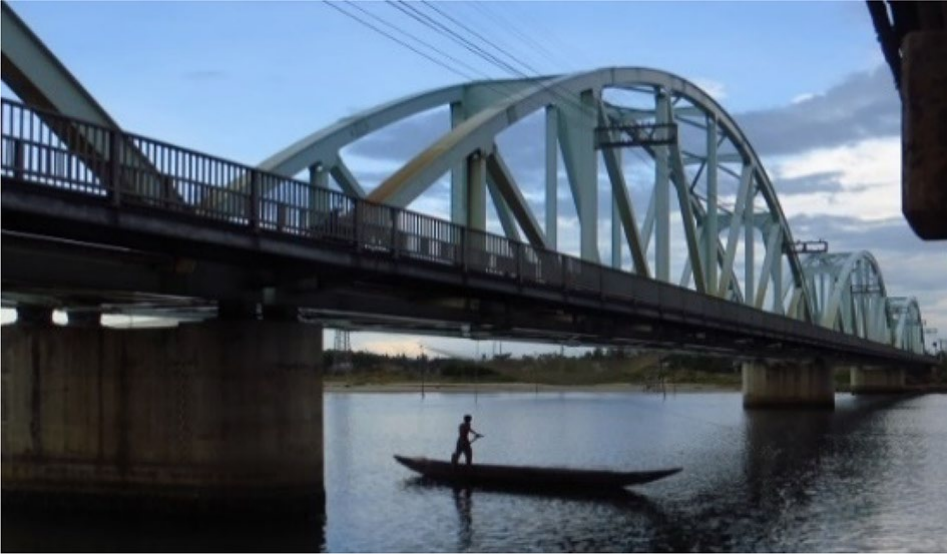
The layout of the fourth span^25^.

Nam O bridge (Fig. 5) is a large-scale truss bridge located in the Da Nang city (in the middle of Viet Nam). The bridge was built in 2011 connecting the most important railway line from the South to the North. The bridge consists of four spans with a length of 75 m for each span. The abutments from Hai Van and Da Nang side are named ${A}_{0}$, and ${A}_{1}$, respectively, whereas three piers, in turn, are named ${P}_{1}$, ${P}_{2}$, ${P}_{3}$.

#### Numerical model

**Figure 6:**
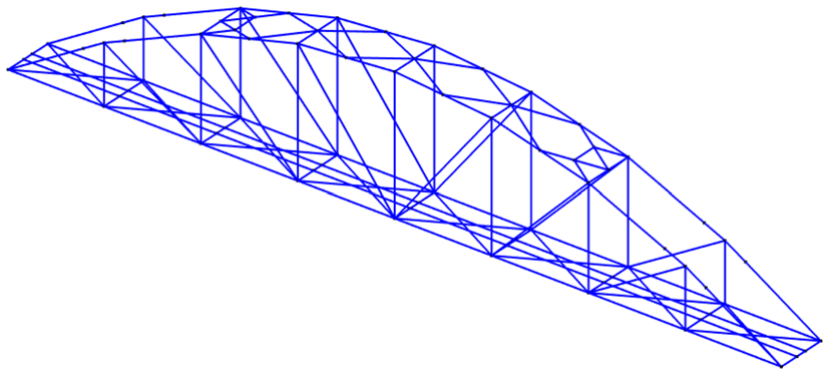
FEM of Nam O bridge^25^.

The FEM model is constructed utilizing MATLAB (Fig. 6). The model consists of 156 elements, and 137 nodes with 356 Degrees of Freedom (DOFs).

#### Measurements

**Figure 7:**
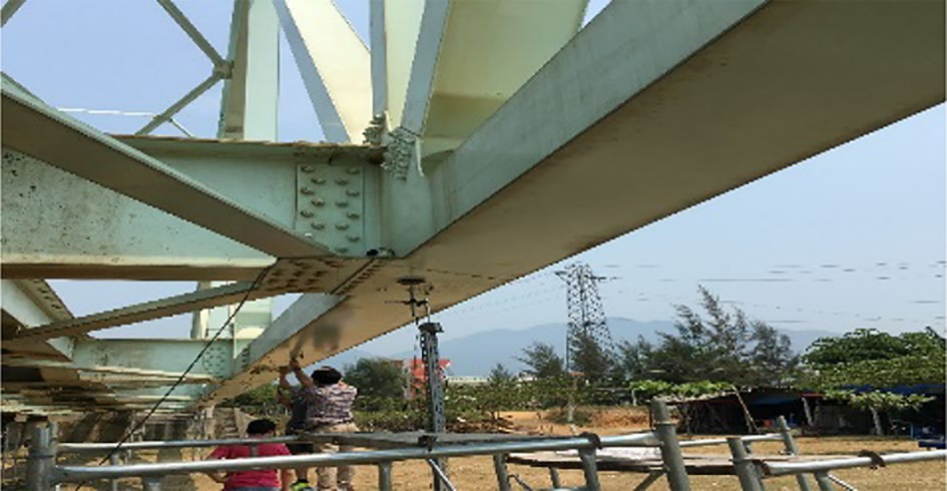
Measuring sensor arrangement^25^.

The measurements were performed on the first span (see Fig. 7). 64 measured nodes were used, in which 40 nodes were fixed, and 24 other ones were roving. More detail about the measurements is described in our previous work^25^.

#### Model updating

In this section, FSPSOSSA is used to deal with the inverse problem of the Nam O bridge. To compare with FSPSOSSA, SSA, ISSA, and other well-known OAs are employed. The parameters used for the considered algorithms are presented in Table 1.

**Table 1:** Parameter values of compared algorithms.

Algorithms	Parameters	Values
PSO	Cognition learning and social learning factor	(${c}_{1}^{^{\prime}}$, ${c}_{2}^{^{\prime}}$) 2.000, 2.000
	Inertia weight $w$	0.900
CS	${p}_{a}$	0.250
BBO	Probability of modifying a habitat	1.000
	Probability limits of immigration	[0 $\div$ 1.000]
	$I$ and $E$	1.000
	Mutation probability	0.005
GA	Type	Real coded
	Crossover	0.800
	Mutation	0.050
MFO	${a}_{1}$	[− 2.000 $\div$ 1.000]
	${b}_{1}$	1.000
GWO	Convergence parameter	Linear reduction [2.000 $\div$ 0]

Uncertain parameters comprise Young's modulus of truss members and the stiffness of bearings^25^. The upper and lower bounds of boundary condition variables are described in Table 2. To reduce the computational time, the stiffness of truss joints is not chosen as an updated variable and its value can be found in our previous work [^25^].

**Table 2:** The boundary condition variables.

Boundary	$E$	$k1$	$k2$	$k3$	$k4$	$k5$	$k6$
Lower	1.9	1.0	1.0	1.0	1.0	1.0	1.0
Upper	2.05	1.6	1.6	1.6	1.6	1.6	1.6

Unit of $k1$,$k2$ ,$k3$, $k4$, is ${10}^{10}$ N/m, unit of $k5$, $k6$ is ${10}^{7}$ N/m, unit of $E$ is ${10}^{5}$ MPa.

The objective function consists of both natural frequencies and mode shapes as shown in Eq. (11):

11 $$\mathrm{\varnothing }={\sum }_{l=1 }^{{n}_{mode}}[1-\frac{\left(\widetilde{ {\varphi }_{l}^{T}}\cdot {\varphi }_{l}\right)2}{ \left({\varphi }_{l}^{T}{\cdot \varphi }_{l}\right)*(\widetilde{{\varphi }_{l}^{T}}*\widetilde{{\varphi }_{l}})}]+{\sum }_{l=1}^{{n}_{mode}}({f}_{l}{-\widetilde{{f}_{l}})}^{2}/{\widetilde{{f}_{l}}}^{2}$$

The first and the second part in the above equation denote the deviation between the first four numerical and measured mode shapes and natural frequencies, respectively. ${\varphi }_{l}$, ${f}_{l}, \widetilde{{\varphi }_{l}}$, $\widetilde{{f}_{l}}$, in turn, are numerical and experimental mode shapes and natural frequencies; “$l$” is the modal order; $T$ is a transposed matrix; ${n}_{mode}$ is the number of considered modes. Table 3 shows natural frequencies of the first four modes. For more detail about the numerical model and measurement of Nam O bridge, the readers are referred to^25^.

**Table 3:** The first four natural frequencies of the bridge.

Modes	Before model updating (Hz)	After model updating (FSPSOSSA)	Measurement (Hz)	Mode type
1	1.47 (1.38%)	1.45 (0%)	1.45	First lateral
2	3.14 (0.96%)	3.11 (0%)	3.11	First torsion
3	3.32 (1.22%)	3.28 (0%)	3.28	Second lateral
4	4.80 (3.90%)	4.54 (1.63%)	4.62	First vertical bending

#### Analysis of convergence level

**Figure 8:**
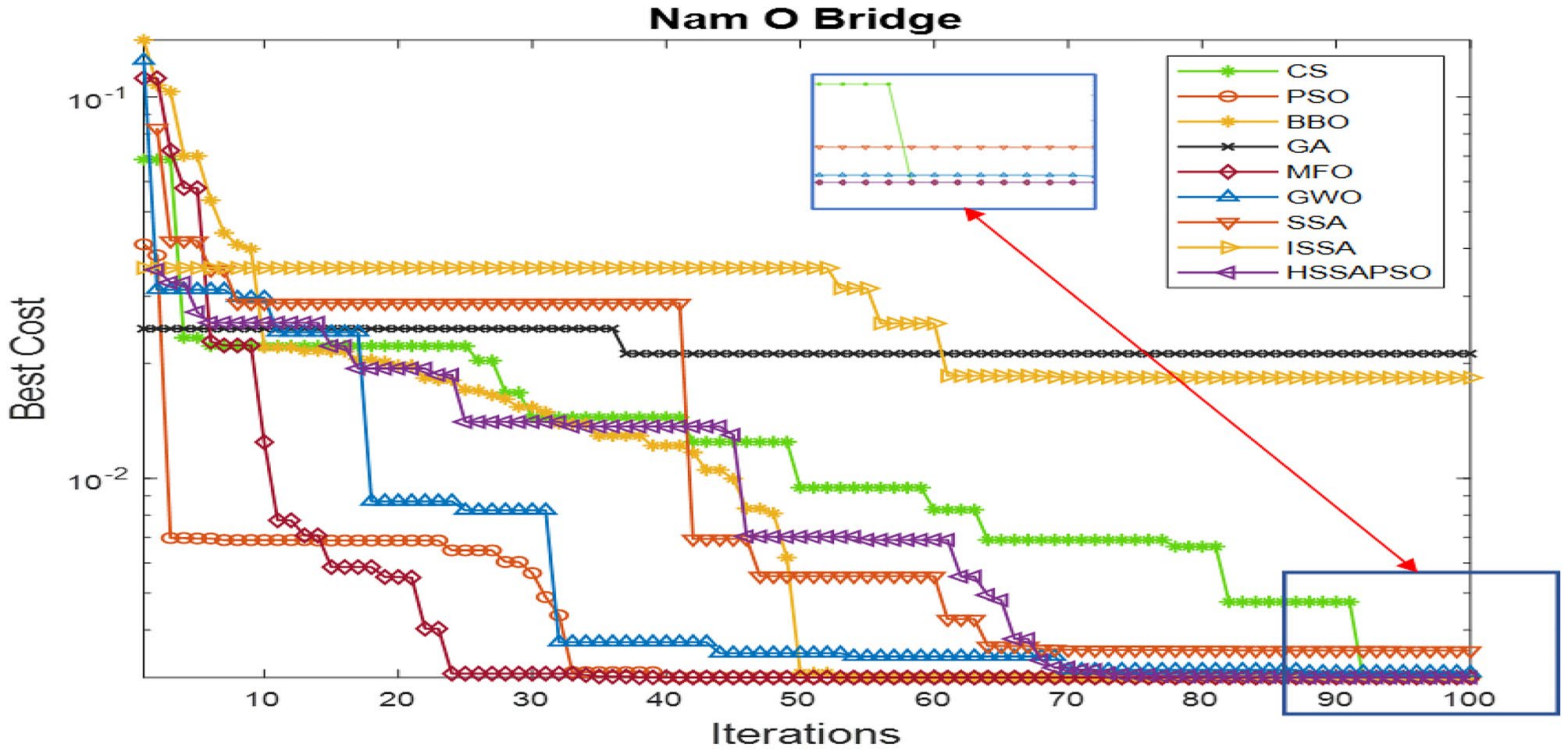
Convergence level.

The condition to complete the algorithm is that the number of iterations reaches 100 steps or the deviation of the objective between the numerical model and measurement is less than ${10}^{-5}$. The convergence level is shown in Fig. 8.

Figure 8 shows a low convergence of GA, ISSA, PSO, and CS. The reason is that these algorithms converge too early, making them difficult to reach optimal solutions. Although the convergence speed of FSPSOSSA is slower than other algorithms at the first steps, with the optimal capacity of the FS combined with the global search capacity of PSO, FSPSOSSA still provides a higher level of convergence than other algorithms.

#### Consideration for accuracy and computational time

To consider the accuracy of the considered algorithms, three values consisting of Mean ($\overline{x }$), Standard Deviation (SD), and Standard Error (SE) are employed. $\overline{x }$ is the average of $N$ samples of the best-obtained values. $\overline{x }$ is calculated based on Eq. (12):

12 $$\overline{x }=\frac{1}{S}{\sum }_{i=1}^{S}{A}_{i}$$

- ${A}_{i}$: The value of sample *i*th
- $$S$$: Sample size
- $\overline{x }$ : mean of $N$ samples.

SD is to measure the amount of variation of a set of values. SD is determined based on Eq. (13):

13 $$SD=\sqrt{\frac{1}{S-1}{\sum }_{i=1}^{S}\left|{A}_{i}-\overline{x }\right|}$$

SE is a statistical term that represents SD of its sampling distribution. SE is determined based on Eq. (14)

14 $$SE=\frac{SD}{\sqrt{S}}$$

The accuracy and computational cost of the considered algorithms are shown in Table 4.

**Table 4:** The accuracy and the computational time of the considered algorithms.

Algorithm	Best	$\overline{x }$	SD	SE	Time (seconds)
CS	0.005100	0.006370	0.001700	0.000310	5643.322100
PSO	0.005100	0.008520	0.001470	0.000268	5524.106200
BBO	**0.003002**	0.004046	0.002622	0.000479	5599.374000
GA	0.021600	0.049670	0.028300	0.004950	7572.460000
DE	0.021194	0.048569	0.026217	0.004787	2973.195700
MFO	**0.003002**	0.004162	0.001803	0.000329	2997.610400
GWO	0.003088	0.006661	0.002920	0.000533	3077.771000
SSA	0.003533	0.021942	0.009980	0.001822	3072.911700
ISSA	0.018333	0.028789	0.002819	0.000515	5571.815000
FSPSOSSA	**0.003002**	**0.003030**	**0.000100**	**0.000018**	**795.593450**

Based on four values including Best, $\overline{x }$, SD, and SE, it is clear that FSPSOSSA is superior to all comparative algorithms. This means that FSPSOSSA provides the best correspondence between the numerical model and measurements of the Nam O bridge. In terms of computational cost, BBO, ISSA, and especially, GA spend a large amount of time, 5599.37 s, 6325.69 s, and 7572.46 s, respectively to complete 100 iterations. In contrast, FSPSOSSA only expends the least computational time (only 795.59 s) for this process since this algorithm uses the vectorization technique to reduce the size of data and the superscalar processor technique to run iterations in parallel. The variables before and after updating are presented in Table 5.

**Table 5:** Result of variables before and after updating. The Units of those variables are similar to those of Table 2.

	$E$	$k1$	$k2$	$k3$	$k4$	$k5$	$k6$
Before	2.0	1.30	1.30	1.30	1.30	1.50	1.50
After (FSPSOSSA)	1.98	1.17	1.16	1.21	1.23	1.35	1.45

## Conclusions and future research

This paper proposes a promising approach to the application of OAs to deal with real-world problems, especially, SHM for a real large-scale truss bridge. To achieve this goal, two targets including accuracy and computational time need to be dealt with. In terms of accuracy, first, we come up with workable solutions to the shortcomings of traditional SSA. This solution includes two main characteristics: balancing the exploration and exploitation capacity and employing the global search capacity of PSO. On the other hand, we exploit the optimal potential of FS to boost the efficacy of OAs. Regarding computational time, up-to-date computing techniques including superscalar processor and vectorization techniques are employed. To compare with FSPSOSSA, other well-known algorithms are also employed. Based on the obtained results, some remarks can be made.

- After model updating, a good agreement between numerical model and FEM is achieved. The biggest deviation between numerical and measured natural frequencies is lower than 10%.
- FSPSOSSA is not only completely superior to SSA, but also surpasses other comparative algorithms in terms of accuracy and computational cost.
- FS is enormous potential to apply for OAs using thresholds such as SSA.
- Apart from improving the accuracy of SSA algorithms in particular and OAs in general, this paper employs up-to-date techniques such as superscalar processor and vectorization techniques for OAs. As a result, the computational time reduces extremely. Hence, this approach is a high potential for SHM of large-scale structures as well as for other real-world problems.
- Further research should conduct to apply the capacity of the proposed approach to detect damages in real applications.

## Data Availability

The datasets used and/or analysed during the current study available from the corresponding author on reasonable request.
